# Acute Rhinitis (Acute Nasal Catarrh)

**Published:** 1912-08

**Authors:** 


### Acute Rhinitis (Acute Nasal Catarrh).

Catarrh is now universally regarded as an acute mixed infection of the nasal membrane, due to a number of bacilli, of which the chief is no doubt the influenza bacillus, especially in the epidemic cases. The membrane suffers a lowering of its resistance by reason of cold, irritation, over-fatigue, excessive drinking, over-eating, and what-not, and offers a field of invasion to the germs. There are three well-marked stages, corresponding to those of every bacterial disease: (1) the stage of invasion, characterized by heat, dryness and swelling of the membrane; (2) the stage of muco-serous exudation, characterized by profuse discharge of muco-purulent exudate; (3) the stage of resolution, in which the discharge gradually diminishes and the membrane resumes its healthy condition.

*Treatment.*—At first small, frequently-repeated doses of tincture of aconite, gtt. j. in a teaspoonful of water. Douche the nasal passages thoroughly with straight Listerine, every hour; at the same time washing out the mouth and rinsing the throat with a 50 per cent solution of the same. This treatment at the outset, accompanied by complete rest in bed, will often abort the attack in twenty-four hours.

The bowels to be moved by a sharp saline purge.

When the nasal flux is well established, no internal remedies surpass ipecac and salicylate of sodium, which may be given in camphor water, as follows:

Vini ipecac.........................Min. ijss
Sodii salicyl.........................gr. xv
Aquae camph.........................oz. jss

To be taken at bed-time.

In this stage of the complaint the nasal douche should be markedly alkaline, and administered warm.

Listerine.........................oz. ss
Sodii bicarb.........................gr. x
Aquae destill.........................oz. ss

Douche, warm, every two hours.

In the third, recuperative stage, 1-grain doses of quinine, and resume douching with full strength of Listerine, every three hours. When patient is obliged to go out in cold air, spray the nose with liquid albolene to protect the membranes.

During an acute attack no surgical measures should be undertaken on the nose, but on the subsidence of the attack look well for spurs, enlarged turbinated bones, etc., and correct any such abnormalities surgically as a prophylactic against future troubles.

